# Supplementary material for: Young men’s everyday life experiences with contraception and unintended pregnancy in Papua New Guinea
Source: PLOS Glob Public Health. 2024 Dec 2;4(12):e0003925. doi: 10.1371/journal.pgph.0003925 (PMC11611134; doi:10.1371/journal.pgph.0003925)
Supplement: S1 File — (DOCX) [file pgph.0003925.s001.docx]

**GUIDE 1 of 2: Adolescent pregnancy in PNG**

**Young men – ever got someone pregnant**

1. Tell me a bit about you and your family? Age? Home? Education? Work? Hobbies? Hopes for future?

**COMMUNITY PERCEPTIONS**

1. What expectations are there of young women and men in this community? (household chores, education, employment, marriage, having children? Differ by gender?
2. When is it okay for young women and me to start having sexual relationships?
3. When is it okay for young women and men to have a child? Age? Marital status? Education?
4. How is having a baby when not married viewed by others (parents, peers, relatives etc.)?

**RELATIONSHIP EXPERIENCE**

1. Can you tell me a bit about your mother of your child?

- Currently in a relationship with that person? How and where did you meet? How many children do you have together? Do you live together? What do your family say or think about this relationship? How have you marked your relationship? What are the good things and bad things about your relationship?

1. Before meeting X, did you have other relationships?

- How long did that/those relationships last? Why or how did it/they end? Did you fall pregnant/have any children together? In what way were they the same or different from your current relationship?

**EXPERIENCES OF PREGNANCY**

1. Can you tell me about the time when you found out your wife/girlfriend was pregnant?

- How did you react/feel? What thoughts/concerns/fears did you have when you found out?
- How old were you?
- Was the pregnancy planned?
- How did you confirm that she was pregnant? (take a test, see a doctor)?
- Who do you turn to/where did you go for support when you found out she was pregnant? Together?
- When did you tell people she was pregnant? Who did you tell? (partner, siblings, parents etc? What was their response? How did telling them that you were pregnant change your relationship with them?
- What conversations did you have with the mother of your child about the pregnancy? What decisions did you make? Together? Alone? Did you face any challenges while making the decision? What impact did it have?
- In what ways did finding out she was pregnant change your life at that time?
- If discontinued pregnancy: What method did you use to discontinue the pregnancy? Why? Where did you go? Why? [e.g. cost, acceptability, accessibility)? Did you discuss discontinuing the pregnancy with anyone? Who? Were there any consequences? [Successful? Unsuccessful? Health, social, cultural, relational?]

1. Can you tell me a bit about your experiences during this pregnancy?

- What were some of the challenges you faced? (access to reproductive services, relationships, schooling, economic etc.)? How were you treated? (by parent, peers and partner)? How did it impact your life? (e.g. schooling, work, relationships)
- Who did you turn to for advice/support during the pregnancy?
- How did you support your partner during the pregnancy? (emotion, practical, financial etc.)
- What involvement did you have during the pregnancy? (emotion, practical, financial etc.)
- What reproductive health services were available to you and the mother during the pregnancy? Did either of you use these services? Were the experiences good or bad? Why? Were there any barriers to you using these services?
- How many months pregnant was your partner when she first visited a doctor/had her first antenatal check-up? Did you go? Why? Why not?
- Where was your baby born? (Hospital, health centre, at home etc? Were you there? What support did you offer? Was your experience of care during the delivery and straight after your baby was born good or bad? Why?

**EXPERIENCES OF FATHERHOOD**

1. Can you tell me a bit about your experiences of being a father?

- How does it make you feel? How has your life changed? How are you treated (by family, partner, peers, community etc.)? What aspects of being a father make you happy? What aspects of being a father are challenging or difficult?
- What effect has having a child had on your life? (social, education, relationships, economic, physical etc.).
- If unmarried: Has having a baby when you are not married created any particular challenges for you or the mother of your child?
- If you need any parenting advice/support who do you turn to?
- What involvement have you had in your child’s life so far? (emotion, practical, financial etc.)
- How do you support your partner to raise your child? (emotion, practical, financial etc.)
- What kinds of expectations do others place on you now that you are a father? (partner, family, relatives, friends etc.)

**SRMH PRACTICES AND SUPPORT**

1. Can you tell me a bit about your sexual history? How old were you the first time you had sex? How many different people have you had sex with?
2. Before your partner got pregnant:

- Did you know about ways to prevent pregnancy? (e.g. contraception, where to access it etc.)
- Where did you learn this information?
- Did you ever talk to anyone about contraceptive use? Who? (partner, friends, family, school or health service etc.)
- What do people say about methods used to prevent pregnancy? Good/bad things?
- Did you use any birth control method to avoid pregnancy? If yes, what kind? (pill, condoms, withdrawal etc.)
- Did you and your partner ever discuss contraceptive use? Whose decision was it to use/not use birth control?

1. If currently sexual active: Are you using contraception? What type of contraception are you using? Where did you get it from? What has influenced this decision?
2. Would you like to have other children? If yes, when? Under which circumstances? Married?
3. Do you think there is sufficient information about sexual and reproductive health accessible to young men and women of your age in this community? What information is currently available? What information is currently missing? What do you think could be done to improve access to information? Why? How would this help?
4. What services are available for SRMH? What services are currently available? What services are currently missing?
5. Which services do you use? Which ones do you not use? Why?
6. What aspects of service delivery could be improved? Why? How would this help?

**CONCLUDING THE INTERVIEW**

- What advice would you give another young person who experienced an unplanned pregnancy?
- Is there anything you would like to add or elaborate on?
- Is there any issue that you feel we haven’t covered in this interview that is important to you that you would like to talk about?

**GUIDE 2 of 2: Adolescent pregnancy in PNG**

**Young men – never got someone pregnant**

1. Can you tell me a bit about you and your family? Age? Home? Education? Work? Hobbies? Hopes for the future?

**COMMUNITY PERCEPTIONS**

1. What expectations are there of young women and men in this community? (household chores, education, employment, marriage, having children? Differ by gender?
2. When is it okay for young women and me to start having sexual relationships?
3. When is it okay for young women and men to have a child? Age? Marital status? Education?
4. How is having a baby when not married viewed by others (parents, peers, relatives etc.)?

**RELATIONSHIP EXPERIENCE**

1. Can you tell me a bit about your girlfriend, wife?

- Currently in a relationship with that person? How and where did you meet? How many children do you have together? Do you live together? What do your family say or think about this relationship? How have you marked your relationship? What are the good things and bad things about your relationship?

1. Before meeting your current girlfriend/wife, did you have other relationships?

- How long did that/those relationships last? Why or how did it/they end? Did you fall pregnant/have any children together? In what way were they the same or different from your current relationship?

**PERCEPTIONS OF PREGNANCY**

1. Can you tell me about pregnancy among young people here?

- What happens when a young man gets a young woman pregnant here? If he is married/unmarried? If it is planned/unplanned?
- How do young men feel when they get someone pregnant? What thoughts/concerns/fears do they have? What about young women?
- Who do young men turn to/where do they go for support when they find out they have got someone pregnant? What about young women?
- How long do young men usually wait before they tell people about the pregnancy? Who do they normal tell? (partner, siblings, parents etc.) How do these people usually respond? Does the pregnancy change their relationship with these people? What about young women?
- Do young men talk to the mother of their babies about their pregnancy? What kinds of things do they talk about/decisions do they make? Challenges faced while making decisions? What impact? What happens if there are disagreements?
- In case of an unwanted pregnancy, what do young men and women do? Discontinue or continue? Why? What method do people use to discontinue a pregnancy? Why? Where do they go? Why? [e.g. cost, acceptability, accessibility] What are the consequences? [Successful? Unsuccessful? Health, social, cultural, relational?]
- Which young people are more likely to get pregnant here? Why? (ages, contraceptive use, lack of information, lack of family support, alcohol use etc.)
- What kinds of things make some young people here more likely to get pregnant than others? Why? (contraceptive use, lack of information, lack of family support, alcohol use etc.)

1. What is it like for young men during the pregnancy?

- What are some of the challenges he might face? (access to reproductive services, relationships, schooling, economic etc? How are they treated? (by parent, peers and partner)? How does it impact their life? (e.g. schooling, work, relationships)
- Who do they turn to for advice/support during their pregnancy?
- Do young men or women use local reproductive health services during their pregnancy here? Why? Why not?
- What involvement does the father of the baby have during their pregnancy? (emotion, practical, financial etc.)

1. And what is it like for young women during the pregnancy?

- What are some of the challenges she might face? (access to reproductive services, relationships, schooling, economic etc? How are they treated? (by parent, peers and partner)? How does it impact their life? (e.g. schooling, work, relationships)
- Who do they turn to for advice/support during their pregnancy?

**PERCEPTIONS OF FATHERHOOD (never had children)**

1. What is it like for young men once their baby is born?

- Can you tell me a story about a young man in the community who got a girl pregnant and had a child?
- What involvement does a boyfriend/husband/father of the baby usually have in the child’s life? (emotion, practical, financial etc.)
- How does it change their lives? (social, education, marriage prospects, economic, physical etc.). How are they treated (by family, partner, peers, community etc.)? What are some of the challenges they face?
- What about if they are unmarried? Is there any additional challenges?
- If young men or their partners need any parenting advice/support who do they turn to?
- What kinds of expectations do others place on young fathers? (partner, family, relatives, friends etc.)? What about young mothers?

**SRMH PRACTICES AND SUPPORT**

1. Can you tell me a bit about your sexual history? How old were you the first time you had sex? How many different people have you had sex with?
2. What do you/young people do to prevent pregnancy? (e.g. contraception, where to access it etc.) Where did you learn this information? Have you ever talk to anyone about contraceptive use? Who? (partner, friends, family, school or health service etc.) What do young people say about methods used to prevent pregnancy? Good/bad things?
3. If sexually active: Do you use any birth control method to avoid pregnancy? If yes, what kind? (pill, condoms, withdrawal etc.) Where did you get it from? What has influenced this decision? Do you ever discuss contraceptive use with your partner? Whose decision was it to use/not use birth control?
4. Would you like to have children? If yes, when?
5. Do you think there is sufficient information about sexual and reproductive health accessible to young men and young women of your age in this community? What information is currently available? What information is currently missing? What do you think could be done to improve access to information? Why? How would this help?
6. What services are available for SRMH? What services are currently available? What services are currently missing?
7. Which services do you use? Which ones do you not use? Why?
8. What aspects of service delivery could be improved? Why? How would this help?

**CONCLUDING THE INTERVIEW**

- What advice would you give another young person who experienced an unplanned pregnancy?
- Is there anything you would like to add or elaborate on?
- Is there any issue that you feel we haven’t covered in this interview that is important to you that you would like to talk about?
