# Supplementary material for: Young men’s everyday life experiences with contraception and unintended pregnancy in Papua New Guinea
Source: PLOS Glob Public Health. 2024 Dec 2;4(12):e0003925. doi: 10.1371/journal.pgph.0003925 (PMC11611134; doi:10.1371/journal.pgph.0003925)
Supplement: Attachment — Submitted filename: Response to reviewers.docx [file pgph.0003925.s003.docx]

**Journal requirements**

| Please include a complete copy of PLOS’ questionnaire on inclusivity in global research in your revised manuscript. Our policy for research in this area aims to improve transparency in the reporting of research performed outside of researchers’ own country or community. The policy applies to researchers who have travelled to a different country to conduct research, research with Indigenous populations or their lands, and research on cultural artefacts. The questionnaire can also be requested at the journal’s discretion for any other submissions, even if these conditions are not met. Please find more information on the policy and a link to download a blank copy of the questionnaire here: <https://journals.plos.org/globalpublichealth/s/best-practices-in-research-reporting>. Please upload a completed version of your questionnaire as Supporting Information when you resubmit your manuscript. | Completed and submitted. |
| --- | --- |
| The [PLOS Data policy](https://journals.plos.org/globalpublichealth/s/data-availability) requires authors to make all data underlying the findings described in their manuscript fully available without restriction, with rare exception. The data should be provided as part of the manuscript or its supporting information, or deposited to a public repository. For example, in addition to summary statistics, the data points behind means, medians and variance measures should be available. If there are restrictions on publicly sharing data—e.g. participant privacy or use of data from a third party—those must be specified. | We have added the following data availability statement:  “Qualitative data collected as part of this research is available as quotes within the paper only. We are unable to make the full data sets available as this was not specified in participant informed consent or the original ethics approval.”  Restricted access to original qualitative data sets is not often permitted by ethical committees, as noted in other recently published papers published in PLOS GPH:   - <https://journals.plos.org/globalpublichealth/article?id=10.1371/journal.pgph.0002846#sec024> - file:///Users/stephen.bell/Downloads/journal.pgph.0000058.pdf - <https://journals.plos.org/globalpublichealth/article?id=10.1371/journal.pgph.0001848> |

**Response to reviewers**

We thank reviewer 1 for their feedback on this review, and appreciate the constructive ideas for improving this work. Please see specific responses to feedback below.

Reviewer 1

| 1 | This paper provides findings from an interesting, large (for qualitative) sample of sexually active men in Papua New Guinea. | Thank you. |
| --- | --- | --- |
| 2 | The introduction promises interesting inquiries into topics and contexts that do not end up being presented in the paper. Given the size of the sample and the purported goal of the research, too much is left unsaid. | This is 1 of 6 outputs from this study that are either published or under review. In combination these unpack the complex social contexts within which unintended pregnancy among young people occurs. It is normal for findings from large qualitative studies to be published across multiple outputs.  We present one clear, focussed aim for this paper (lines 107-109): “this paper aims to understand their use of modern and other contraceptives in the context of pregnancy prevention and unintended pregnancy.”  We feel this is achieved in this paper, in a form that is appropriate for a short public health paper. We pack a large amount of insight about how young men’s experiences with contraception leads to unintended pregnancy (see lines 52-61 of the abstract; and the extensive data and analysis presented through lines 171-478 in the findings and discussion sections), based on rigorous inductive analyses of this qualitative data set. |
| 3 | The main conclusions presented here do not break new ground | While complementing findings from other research in international settings, we feel strongly that this paper does break new ground both nationally in PNG and at a regional level in Asia and the Pacific: (1) there is extremely limited data about the use of modern contraceptives *among young men* in Asia and the Pacific, and in the introduction (lines 94-105), we make the case as to why it is important, but rare, to focus SRH research on young men; (2) Without data specific to national settings, generated through rigorous scientific research, and published in high quality academic journals, it can be hard to advocate for the need for greater investment in efforts to engage young men in SRH policies and practices that protect young women. This is a major knowledge gap inhibiting progressive PNG adolescent SRH policy. |
| 4 | The main conclusions presented here … do [not] highlight the specific contexts that are alluded to on line 89 (“complex social, cultural and religious contexts.”) | We have revised this statement to focus on just the *social* contexts within which unintended pregnancy occurs (see line 80); this now aligns more strongly with the discussion and conclusions. |
| 5 | The introduction refers to the way young men relate to pregnancies in ways that are “social, relational, gendered, and emotional” (line 98) but the main themes introduced do not get there either. | Thank you so much for this very useful feedback. This statement was included primarily to define that young men also ‘experience pregnancy’, but just in ways that differ to young women (i.e. in non-biological ways). However, we see the opportunity that you raise here, and as such have reworked the paragraph in the introduction (see lines 94-105); in accordance with the specific aim of this paper (see note 2 above), we have also reworked the discussion (lines 388-478) to more clearly reflect on the findings that are presented and engage with these ideas. |
| 6 | The paper does not identify themes that lead to “mechanisms for change” (line 113) alluded to in the Introduction. | Mechanisms for change are already outlined in detail in lines 496-525. To supplement these, we have also introduced sentences throughout the revised discussion sections to point to clear mechanisms for change there too (see lines 416-419, 435-439, 463-467, and 474-478).  Also see note 12 below. |
| 7 | Insufficient information is provided about the data and the data collection | Revised; see note 13 below. |
| Abstract | | |
| 8 | Provide information on the sample (where they were recruited from). | This has been added (line 53). |
| 9 | There seems to be some circular logic in the major themes listed in the abstract here – low contraceptive uptake is defined by, or precedes, non-use of modern contraceptive methods, and low use, or inconsistent use of condoms, withdrawal, and calendar methods. | This has been revised to more closely replicate the themes reported in the findings section (see lines 56-64). |
| Introduction | | |
| 10 | Compared to US rates (77% 2006-2010, per NCHS) , the % of births among age 15-19 that are reported as unintended here (“more than a quarter,” line 83 – which I guess covers everything from 26 through 100%, but is misleading) is markedly low. If this were in fact the rate, how does this highlight the public health priority for reducing unintended births? How does this relate to the complex social, cultural, and religious contexts (line 89). | Thanks for providing this feedback. The DHS data set is the best available national level data in this setting, but it is inadequate in many respects, particularly with regard to inclusion of unmarried young women in the sampling. This means the survey likely underreports these issues. In light of this feedback, we have restructured the introduction in a way that more convincingly sets up the need for the research we are reporting (see lines 78-92). |
| 11 | In the title, abstract, and the introduction it should be made clear that this is a sample of men from the general population – I think? As noted below, there is inadequate information on from where participants were recruited or sampled and wh. | We have added the term, ‘sampled from the general population’ in the abstract (line 50), and in the opening paragraph of the methods section (line 1119). |
| 12 | While lofty, the claims lines 111-113 that the authors “take a deliberate ethical (18) or values-based (21) stance, located in the desire for social justice – we centre, work with and learn from the stories of young men to identify mechanisms of change required to address inequities that undermine the health and wellbeing of young people” would need to be presented in the Discussion, not the Introduction, and would need to be supported with data and / or methods. | We have removed lines 111-113.  However, we still adhere to these values in this paper. We identify mechanisms of change derived from young people’s lived experiences (see note 6 above). |
|  | Methods | |
| 13 | How were the participants recruited and sampled (beyond purposeful sampling using snowball sampling). Why snowball sampling (especially given the claim, line 435-6 a range of lived experiences)? Provide some rationale for why this method was used – presumably it would not be that hard to find sexually active young men who don’t represent a specific population. | We have provided more detail about the sampling and recruitment of the young men into this study (see lines 139-146).  In PNG (and many other settings in the Asia Pacific), it is extremely hard to undertake rigorous community-based qualitative sexual and reproductive health research with young people who engage in premarital sex because it is forbidden and can lead to punishment, exclusion and discrimination. For this reason, we have provided more detail about the intended vs actual recruitment strategies to illustrate to the reader how complicated it can be to engage young people in this type of deep, rigorous community-based qualitative research. |
| Findings | | |
| 14 | As mentioned above – the intro and discussion talk about nuanced concepts (sexual debut, for example) that are not captured in the themes, which seem very one-dimensional (e.g. “not using condoms during early sexual experiences.”) | See comment against note 4 above.  In general we have presented the thematic analysis of the data in the findings section, and pull together the main themes introduced in the introduction with reflections on the analysis in the discussion section. This is a common approach to writing qualitative papers for public health audiences.  However, in light of your feedback, we have made two changes: (1) we have added more signposts to the social themes arising in the analysis throughout the findings section; and (2) restructured the discussion section.  We hope these changes strengthen the paper. |
| 15 | Line 190-192 presents a very rich example – where the respondent discusses having sex with a partner who is already pregnant – is the participant indicating that his understanding is that she got pregnant through sexual activity that he does not consider having “sex”? | Lines 190-192 in the first draft relate to lines 198-202 in the revised draft. We have revised the text here to clarify the meaning of the quote: that after being in a relationship for 4 years (but not having sexual intercourse), they had sex for the first time and fell pregnant doing so, because they did not know about contraceptives. |
| 16 | Difficulties accessing condoms, for instance – this could intersect quite a bit with social and cultural and religious themes that were referred to in the Intro, but it does not delve into any depth. | See notes 4 and 14 above regarding the refocus on the social contexts of contraceptive use, and the changes made throughout the findings and discussion sections. |
| 17 | Given the amount of data, tables could be used to present more data with more in-depth exploration of the quotes. | The current presentation of data is preferred. We specifically wanted to centre the voices of young people in the paper; removal of data from the main text into tables does not support centring of participants’ lived experience in journal publications as it enables readers to avoid the quotes.  We have developed the discussion further to build more in-depth exploration of the data presented; we have also reworked the findings in response to points 14-15 above. We hope this is has strengthened the paper overall. |
| 17 | The data collection instrument/interview guide should be provided as an appendix. | This has been added as supplementary information (in English and Tok Pisin). |
| Implications for Policy and Practice | | |
| 18 | The implications follow from the findings, but they don’t bring into the discussion the specific context of PNG. | The policy and practice section specifically discusses the implications of the research findings for policy and practice in PNG. We have added some text into this section so it is clear for the reader the focus is on PNG. These implications were co-developed with the PNG co-authors and research partners, and were perceived as identifying some core opportunities for improving policy and practice. |
