# Supplementary material for: Young men’s everyday life experiences with contraception and unintended pregnancy in Papua New Guinea
Source: PLOS Glob Public Health. 2024 Dec 2;4(12):e0003925. doi: 10.1371/journal.pgph.0003925 (PMC11611134; doi:10.1371/journal.pgph.0003925)
Supplement: Attachment — Submitted filename: Dear authors.docx [file pgph.0003925.s004.docx]

**Dear authors:**

Your manuscript is well-organized and covers important aspects of the study. Here are few comments and suggestions to enhance clarity and impact:

Title: -

- The title is clear and descriptive. It effectively conveys the focus of the study, however the title could be more specific focusing on the core aim of the study, such as understanding the barriers to use or specific settings where challenges are most prevalent. And it is possible to shorten the title as: *Understanding Young Men’s Use of Contraceptives in Papua New Guinea*

**Abstract: -**

**Introduction:** it could be better if a gap statement is included

**Methodology: it will be good if it is written like this:** This qualitative study involved semi-structured interviews with 35 sexually active young men aged 15-24 years, who were purposively sampled from the general population within community-based settings. The data were analyzed using inductive thematic analysis.

**Discussion (line 390-401)**

- The discussions are detailed and informative. Breaking down long sentences into shorter ones can improve readability. For example:
- “Our analysis of young men’s everyday experiences of using condoms and other contraceptives highlights clear drivers of unintended adolescent pregnancies. These include non-use of any modern method at first sex or during early sexual experiences, inconsistent use of condoms (often only after first pregnancy experiences), and difficulties accessing condoms from health service providers, pharmacies, and stores.”
- Ensure consistent use of semicolons and commas when listing items

**Conclusion (line 540-543)**

- The conclusions section is strong but could be more impactful with a clear statement on the implications of your findings. For example:
- “Young men’s everyday stories provide a unique lens through which we can identify mechanisms of change required to address the health and social inequities associated with unintended pregnancy among young men and young women in PNG and beyond. These findings suggest the need for targeted interventions that address the specific barriers young men face in accessing and using contraceptives effectively.”

Overall, your manuscript is well-written and covers significant aspects of the study. These minor adjustments should help improve clarity and impact
