# Supplementary material for: Young men’s everyday life experiences with contraception and unintended pregnancy in Papua New Guinea
Source: PLOS Glob Public Health. 2024 Dec 2;4(12):e0003925. doi: 10.1371/journal.pgph.0003925 (PMC11611134; doi:10.1371/journal.pgph.0003925)
Supplement: Attachment — Submitted filename: Response to reviewers 2.docx [file pgph.0003925.s005.docx]

**Journal requirements**

Please include a complete copy of PLOS’ questionnaire on inclusivity in global research in your revised manuscript. Our policy for research in this area aims to improve transparency in the reporting of research performed outside of researchers’ own country or community. The policy applies to researchers who have travelled to a different country to conduct research, research with Indigenous populations or their lands, and research on cultural artefacts. The questionnaire can also be requested at the journal’s discretion for any other submissions, even if these conditions are not met. Please find more information on the policy and a link to download a blank copy of the questionnaire here: <https://journals.plos.org/globalpublichealth/s/best-practices-in-research-reporting>. Please upload a completed version of your questionnaire as Supporting Information when you resubmit your manuscript.

Completed and submitted after the first review.

Additionally, please include a detailed discussion of the 5 other publications that are related to this manuscript with citations where possible. In this discussion please include a scientific rationale for separating the study outputs into 6 manuscripts and detail how they differ.

We have an extensive data set which we are using to answer the following study aims and objectives, as funded by NHMRC:

**Aim:** Using qualitative methods, we will examine the experiences of adolescent pregnancy among married and unmarried Papua New Guineans aged 15-24 years. The overall goal will be to guide the development of youth-centred health promotion strategies to prevent and mitigate the impact of adolescent pregnancy and associated adverse health outcomes. Specific objectives will be to:

1. Document young women’s and men’s perceptions and experiences of pregnancy;

2. Examine young people’s socio-sexual behaviours, and the social, religious, cultural and service-based contexts that influence risk of adolescent pregnancy and adverse health outcomes;

3. Analyse the strategies young people use to prevent or mitigate the impact of pregnancy;

4. Identify successes and gaps in government and community responses to adolescent pregnancy;

5. Develop context-appropriate health promotion service- and community-based strategies to prevent and mitigate the impact of adolescent pregnancy.

The papers that have been published or are in progress are detailed below, for your information. As you will appreciate, we have cited papers that are already published already in this paper. But it is not appropriate to refer to work in progress.

- Paper 1: concept paper (published journal article): Bell S, Kennedy E, Black K, Vallely A, Vallely L, Mola G, et al. Youth-centred research to help prevent and mitigate the adverse health and social impacts of pregnancy amongst young Papua New Guineans. Reproductive Health Matters. 2018;26(54):5-12.
- Paper 2: socio-structural determinants of risk of pregnancy among ***very young men aged 15-17 years***, filling important evidence gap among younger cohort who are under-researched (published book chapter): Aeno H, Mitchell E, Ase S, Nake Trumb R, Ofi PS, Kelly-Hanku A, et al. Socio-structural influences on young men’s experiences of sex, pregnancy and pregnancy prevention in Papua New Guinea. In: Kelly-Hanku A, Aggleton P, Malcolm A, editors. Sex and gender in the Pacific: contemporary perspectives on sexuality, gender and health. London: Routledge; 2023. p. 109-22.
- Paper 3: socio-structural determinants of risk of pregnancy among very young women aged 15-17 years, filling important evidence gap among younger cohort who are under-researched (paper under review at BMJ Global Health; Submitted paper title: Socio-structural influences on unintended pregnancy among girls aged 15-17 years in Papua New Guinea
- Paper 4: Paper exploring young peoples abortion trajectories and sexual agency from pre-conception to post abortion (paper under review at Culture, Health & Sexuality); Submitted paper title: Agency in young people’s unsafe abortion trajectories in Papua New Guinea
- Paper 5: Paper examining forms of social and institutional support that can help transition young women to safe abortion and self care in PNG (paper in draft; will submit to one of Lancet GH, Lancet Western Pacific, PLOS GPH or BMJ GH)
- Paper 6: Paper examining young people’s experiences of parenthood (paper in draft; will submit to CHS or Journal of Youth Studies)
- Paper 7: Young people’s engagement in services along the the SRH care continuum (paper in draft; will submit to Health Policy & Planning)

**Response to reviewers**

We thank reviewer for their feedback on this review, and appreciate the constructive ideas for improving this work. Please see specific responses to feedback below.

Reviewer

| 1 | The title is clear and descriptive. It effectively conveys the focus of the study, however the title could be more specific focusing on the core aim of the study, such as understanding the barriers to use or specific settings where challenges are most prevalent. And it is possible to shorten the title as: *Understanding Young Men’s Use of Contraceptives in Papua New Guinea* | Thank you. We have revised this slightly, though wish to keep the broader focus on everyday life experiences clear for future readers. New title is:  Young men’s everyday life experiences with contraception and unintended pregnancy in Papua New Guinea |
| --- | --- | --- |
| 2 | Abstract: **Introduction:** it could be better if a gap statement is included. | Thank you. We have added this. The new statement is:  Unintended adolescent pregnancy is a public health priority in Papua New Guinea (PNG), where national policies specify need for easier access to reliable modern contraceptives. To reduce young people’s experiences of unintended pregnancy in PNG, improved understandings of use of modern and other forms of contraception within young people’s relationships is required to support the development of new sexual and reproductive health (SRH) programs and policies. The aim of this paper is to understand young men’s use of modern and other contraceptives. |
| 3 | Abstract: **Methodology: it will be good if it is written like this:** This qualitative study involved semi-structured interviews with 35 sexually active young men aged 15-24 years, who were purposively sampled from the general population within community-based settings. The data were analyzed using inductive thematic analysis | Thank you. We have revised as suggested:  This qualitative study involved semi-structured interviews with 35 sexually active young men aged 15-24 years, who were sampled purposively from the general population within community-based settings. Data were analysed using inductive thematic analysis techniques. |
| 4 | **Discussion (line 390-401)**  • The discussions are detailed and informative. Breaking down long sentences into shorter ones can improve readability. For example:  • “Our analysis of young men’s everyday experiences of using condoms and other contraceptives highlights clear drivers of unintended adolescent pregnancies. These include non-use of any modern method at first sex or during early sexual experiences, inconsistent use of condoms (often only after first pregnancy experiences), and difficulties accessing condoms from health service providers, pharmacies, and stores.”  • Ensure consistent use of semicolons and commas when listing items | Thanks you for this.  We have been through and edited the discussion section accordingly to enhance ease of reading the paper, while keeping the detail where we can. We hope this clarifies things for you. |
| 5 | The conclusions section is strong but could be more impactful with a clear statement on the implications of your findings. For example:  • “Young men’s everyday stories provide a unique lens through which we can identify mechanisms of change required to address the health and social inequities associated with unintended pregnancy among young men and young women in PNG and beyond. These findings suggest the need for targeted interventions that address the specific barriers young men face in accessing and using contraceptives effectively. | We have revised the conclusions as suggested, bringing this statement to the very end of the paper so it is much clearer. Thanks for this feedback. |
